# Camera-view supervision for bird's-eye-view semantic segmentation

**DOI:** 10.3389/fdata.2024.1431346

**Published:** 2024-11-15

**Authors:** Bowen Yang, LinLin Yu, Feng Chen

**Affiliations:** AI Safety Laboratory, Department of Computer Science, The University of Texas at Dallas, Richardson, TX, United States

**Keywords:** segmentation, perception, autonomous driving (AD), supervision, birds-eye-view, nuScenes dataset

## Abstract

Bird's-eye-view Semantic Segmentation (BEVSS) is a powerful and crucial component of planning and control systems in many autonomous vehicles. Current methods rely on end-to-end learning to train models, leading to indirectly supervised and inaccurate camera-to-BEV projections. We propose a novel method of supervising feature extraction with camera-view depth and segmentation information, which improves the quality of feature extraction and projection in the BEVSS pipeline. Our model, evaluated on the nuScenes dataset, shows a 3.8% improvement in Intersection-over-Union (IoU) for vehicle segmentation and a 30-fold reduction in depth error compared to baselines, while maintaining competitive inference times of 32 FPS. This method offers more accurate and reliable BEVSS for real-time autonomous driving systems. The codes and implementation details and code can be found at https://github.com/bluffish/sucam.

## 1 Introduction

Autonomous driving systems rely on accurate and reliable perception systems to safely navigate the world around them. A widely adopted perception technique is bird's-eye-view semantic segmentation (BEVSS). BEVSS fuses sensor inputs into a top-down, overhead view of a vehicle's surroundings, orthographically mapping key elements such as vehicles, roads, and lanes. A bird's-eye-view (BEV) representation is crucial for autonomous driving systems. It is very useful to assess the surrounding environment and plan safe actions.

Prior approaches to this problem rely on end-to-end learning to learn BEV representations (Hu et al., 2021; Philion and Fidler, 2020; Zhou and Krähenbühl, 2022). A key problem in BEVSS is mapping features from the camera-view perspective to the bird's-eye-view perspective. The geometric relationships between the inputs seen by the camera and the outputs projected in BEV are represented by the relative depth of objects within the scene, so depth perception is crucial. The two primary approaches to BEVSS differ on whether they model depth explicitly or implicitly. Methods such as LSS (Philion and Fidler, 2020) and FIERY (Hu et al., 2021) leverage direct geometric relationships to transform features from camera-view features to BEV. These methods require an explicit camera-view depth estimate in order to project camera-view features to BEV. Other methods, such as CVT (Zhou and Krähenbühl, 2022) and GKT (Chen et al., 2022) leverage attention to directly learn implicit geometric relationships, and thus do not require an explicit depth estimate. However, both of these methods have major shortcomings regarding the quality of camera-view to BEV projections.

In this work, we propose two novel improvements to the traditional BEVSS approach, focusing on the family of methods that explicitly model depth. First, we introduce a supervision process for extracting camera-view features. We do this in order to improve the quality of feature extraction in camera-view, which improves feature relevance in birds-eye-view. In addition, we propose supervising the explicit camera-view depth estimation that is used to map camera features to BEV. Previous BEVSS methods rely on the indirect supervision of depth from the final detection loss. We show that this leads to inaccurate depth mappings, which lowers accuracy. We show that our method significantly improves the camera-view depth estimation, which provides better information about the relative distance and positioning of objects. Through extensive experiments, we show that our BEVSS model with camera-view segmentation supervision and camera-view depth supervision outperforms current methods on real-world benchmarks by improving the quality of feature extraction in camera-view and the reliability of camera-view depth when compared to the baseline end-to-end approach.

To summarize, our main contributions are as follows:

- we propose directly supervising camera-view depth estimation. We first map LiDAR points to the camera-view to generate dense depth labels, and then directly supervise categorical depth estimation using these labels to improve the accuracy and reliability of camera-view depth estimation. The accuracy of the depth prediction on the nuScenes dataset increases by a factor of 30 times, and the BEV segmentation IOU increases by 2.6% from without supervision. Empirical results indicate that this approach significantly enhances the quality of BEVSS, improving the accuracy of camera-to-BEV mapping.
- We propose directly supervising segmentation feature extraction in camera-view. We utilize semantically labeled LiDAR points that are projected to camera-view to supervise relevant feature extraction from the camera-view encoder. Our empirical results demonstrate that adding camera-view segmentation supervision allows our model to learn a more meaningful camera-view representation, achieving 78.9% IOU in camera-view. This improves the quality of BEVSS by a significant margin on the nuScenes dataset, beating the best baseline by 2.7%.
- We demonstrate that our method outperforms the current real-time BEVSS models on the most popular real-world dataset, nuScenes (3.8% IOU improvement), while still only requiring camera RGB inputs at testing time. It runs inference at 32 FPS on an RTX 2080 Ti GPU, which is very competitive with the real-time baselines that we compare to.

## 2 Related work

### 2.1 Bird's eye view semantic segmentation

The goal of BEVSS in autonomous driving is to predict the semantic layout of a scene in the bird's-eye-view perspective. BEVSS is a crucial perception task for downstream applications such as path planning, where an accurate representation of the surroundings is needed. BEVSS relies on transforming sensor inputs, such as camera images or LiDAR point-clouds, into bird's-eye-view. Broadly, there are two common approaches to this task.

Implicit geometric methods leverage learned geometric relationships between camera-view and BEV (Zhou and Krähenbühl, 2022; Chen et al., 2022; Pan et al., 2020; Xu et al., 2022b; Roddick and Cipolla, 2020; Gosala and Valada, 2022). These methods often utilize transformers (Zhou and Krähenbühl, 2022; Liu et al., 2023; Xu et al., 2022b; Roddick and Cipolla, 2020; Gosala and Valada, 2022) or MLPs (Pan et al., 2020) to encode camera-view images to the BEV space. CVT (Zhou and Krähenbühl, 2022) uses a cross-view transformer to learn the camera to BEV transform. BEVFusion (Liu et al., 2023) introduces a fusion mechanism that integrates information from multiple sensors into a common BEV space. CoBEVT (Xu et al., 2022b) introduces a collaborative approach by fusing features from multiple agent vehicles to create a more comprehensive BEV representation.

Explicit geometry based methods focus on directly projecting camera-view features to BEV (Philion and Fidler, 2020; Hu et al., 2021; Roddick et al., 2018; Harley et al., 2022). Methods in this category estimate a depth distribution that is used for projection (Philion and Fidler, 2020; Hu et al., 2021). FIERY (Hu et al., 2021) introduces instance segmentation and temporal modeling in BEV. OFT (Roddick et al., 2018) forgoes the depth distribution to uniformly project features into BEV. SimpleBEV (Harley et al., 2022) utilizes radar aggregation to further improve the camera-to-BEV mapping. Other works explore combining BEV segmentation with other tasks such as object detection to improve the performance (Kumar et al., 2024; Xie et al., 2022).

In previous approaches, whether implicit or explicit, the transform from camera-view to birds-eye-view is learned indirectly, typically through the final segmentation loss. In contrast, we propose supervising the view-transformation by directly learning the camera-view depth distribution during training. Similarly, the feature extraction in camera-view is supervised by the final segmentation loss, whereas in this work we directly supervise it during training. Through experiments we show that directly supervising the camera-view depth estimation and camera-view feature extraction significantly improves the accuracy of the BEV prediction.

### 2.2 3D object detection

3D object detection is a perception task that is commonly in autonomous perception systems. The task is to locate objects of interest in a scene by estimating 3D bounding boxes. Early approaches perform 2D object detection and predict depth information to project to 3D (Manhardt et al., 2019; Girshick et al., 2014). With the popularization of LiDAR and other point-scanning technology, methods that utilize point-cloud data rose to the forefront. PointNet (Qi et al., 2017a) directly processes point-clouds leading to improved object detection. PointNet++ (Qi et al., 2017b) refined this approach further, addressing the hierarchical structure of point clouds. Voxel-based methods such as VoxelNet (Zhou and Tuzel, 2018) convert point-clouds into a voxel grid to leverage 3D convolutions. Pseudo-LiDAR based approaches project image features into 3D by predicting a pseudo point-cloud rather than using LiDAR. This allows these methods to only need camera inputs at testing time. BEVDepth (Li et al., 2023) proposes supervising the point-cloud projection. BEVFusion (Liu et al., 2023) proposes directly fusing LiDAR and camera features in order to incorporate multi-sensor information into 3D object detection.

BEVSS and 3D object detection share significant similarities, particularly in tasks like autonomous driving where understanding complex environments is critical. Both techniques require sensor fusion, integrating data from LiDAR, cameras, and radar to create a comprehensive 3D representation of the scene. BEV segmentation classifies regions (e.g., roads, vehicles), while 3D object detection identifies objects and their spatial boundaries. Both face challenges such as occlusion handling, where parts of the environment are obscured, and the need for real-time processing to ensure fast and accurate decision-making. These shared goals and obstacles make advancements in one field directly applicable to the other.

### 2.3 Monocular depth estimation

Monocular depth estimation tries to infer depth information from a single RGB image. Traditional approaches rely on hand-crafted features and probabilistic graphical models (Saxena et al., 2005, 2008). Deep learning-based methods have demonstrated promising results by learning depth cues directly from data. Eigen et al. (2014) introduced one of the first CNN based models for depth estimation. Some approaches proposed a fully convolutional architecture that improved performance (Laina et al., 2016). Other notable works include the use of conditional random fields (Liu et al., 2015), adversarial training (Chen et al., 2018), and attention mechanisms (Xu et al., 2022a) to enhance depth prediction. Recently, methods using self-supervised (Godard et al., 2019; Liu et al., 2023) and unsupervised (Godard et al., 2017; Sun et al., 2023) approaches rose to prominence, alleviating the need for large-scale ground truth depth data.

Monocular depth estimation plays a crucial role in enhancing BEVSS. In BEVSS, understanding the spatial structure of the environment is vital for accurate scene interpretation, and monocular depth estimation helps bridge the gap between 2D image data and 3D scene representation. By leveraging monocular depth cues, BEVSS can estimate object heights, relative distances, and spatial relationships, which are critical for generating precise top-down views.

## 3 Method

**Figure 1:**
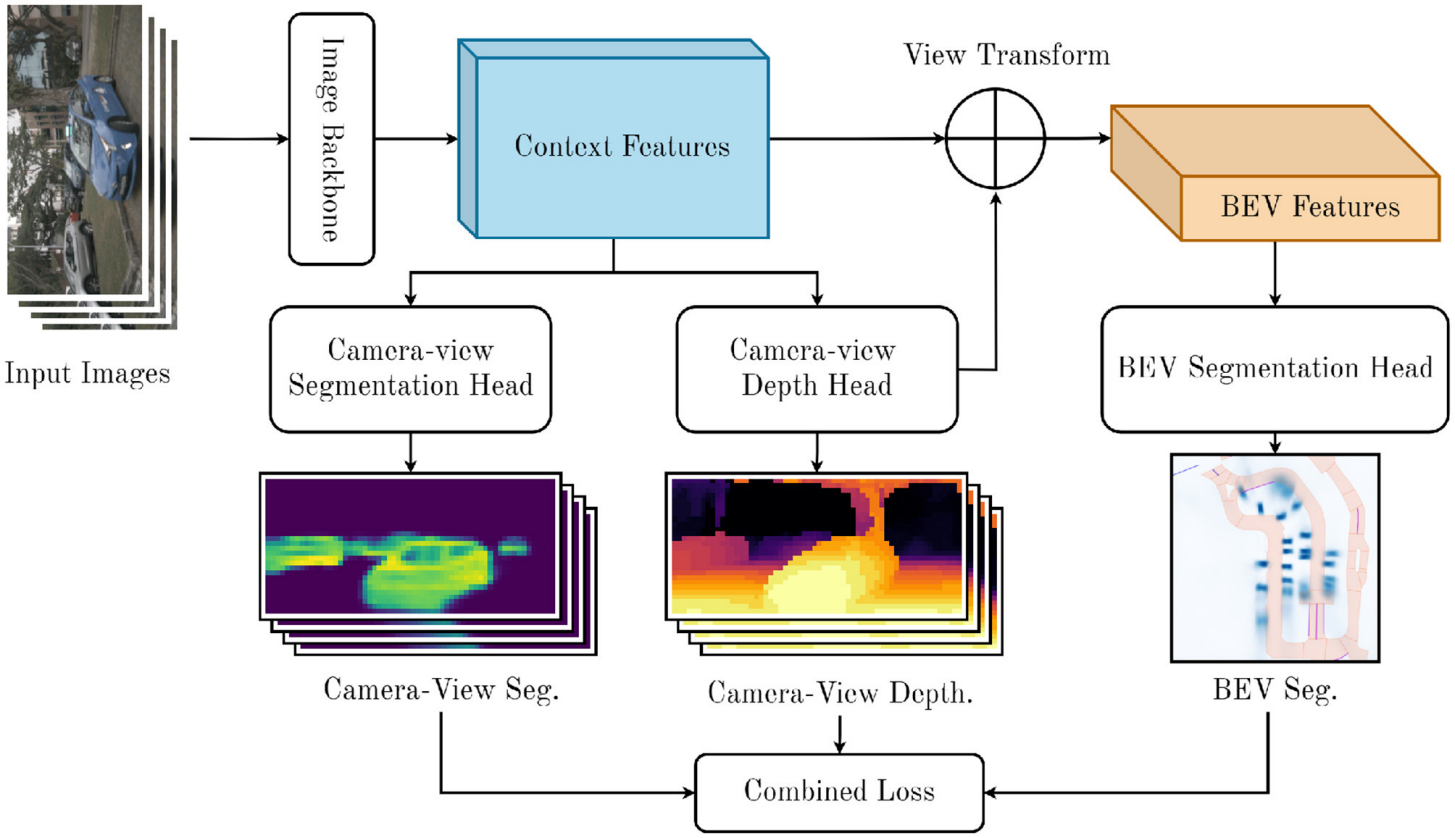
Diagram of our proposed model. We extract context features from input images, predict camera-view segmentation and depth, project camera features into BEV view, and predict a BEV occupancy map.

In this section, we introduce our proposed method for the BEVSS task. The fundamental structure of our model follows LSS (Philion and Fidler, 2020): Given *N* camera-view images ${{\text{X}}_{k}\in {\mathbb{R}}^{3\times H\times W}}_{N}$ with intrinsics ${\text{I}}_{k}\in {\mathbb{R}}^{3\times 3}$ and extrinsics ${\text{E}}_{k}\in {\mathbb{R}}^{3\times 4}$, our goal is to generate a bird's-eye-view representation of the scene. From this, we predict a binary segmentation mask in the BEV frame **y**∈ℝ^*C*×*X*×*Y*^, where *X* and *Y* denote the BEV coordinate dimensions. In the following sections, we will introduce our novel components. We summarize our proposed architecture in Figure 1.

The basic framework of our proposed architecture follows LSS and FIERY (Philion and Fidler, 2020; Hu et al., 2021). There are two stages to our architecture. The first stage extracts features from inputs and projects them into 3D space. The second stage pools 3D features into a BEV voxel grid that we use to predict semantic segmentation.

The first stage of our model extracts context features and estimates a depth distribution for *N* camera-view input images. We generate representations at all possible depths for any given pixel by predicting a probabilistic categorical depth distribution for each pixel. LSS supervises this depth estimate with only the final detection loss. Instead, we propose a novel process to supervise the depth distribution prediction and context feature extraction, which we expand on in Sections 3.1, 3.2. In this context, we consider a set of discrete depths *D* and define |*D*| points {(*h, w, d*)∈ℝ^3^∣*d*∈*D*} at each pixel in the camera-view. For a given pixel *p* in camera-view *M*, we predict a context vector **c**∈ℝ^*C*^ with *C* channels and a depth distribution $\hat{\alpha }\in {△}^{|D|-1}$. The context features are projected into the 3D space by scaling them with the probabilistic depth distribution. The context feature ${\text{c}}_{d}\in {\mathbb{R}}^{C}$ associated with point *p*_*d*_ is then defined as the context vector **c** scaled by the corresponding depth probability ${\hat{\alpha }}_{d}$.


(1) $${\text{c}}_{d}={\hat{\alpha }}_{d}\text{c}.$$

The next stage of our model is voxel pooling, which combines extracted features from camera-view into a unified coordinate system, and pools them into a feature map. We follow the voxel-pooling method described in BEVFusion (Liu et al., 2023). We first associate each point in the camera-view point cloud with a cell in the BEV grid. We can precompute this, since the position for each point in the pointcloud is fixed, unlike in LiDAR. We sort all points according to the grid indices and record the rank of each point, so that all points within the same BEV cell will be consecutive. We aggregate the features in the BEV grid by performing a sum pooling on the features. This creates a *C*×*H*×*W* BEV tensor that can be processed by a standard CNN to create BEV predictions.

### 3.1 Depth supervision

In previous models, the supervision of depth distribution relied solely on the final detection loss, which limited their ability to effectively capture depth information. To address this limitation, we introduce direct supervision for discrete depth prediction from the camera-view perspective. This camera-view depth supervision allows our model to better capture spatial relationships and depth cues, resulting in more accurate camera-to-BEV mappings.

To generate dense ground-truth depth information, we use LiDAR point clouds. A LiDAR point *P* = (*X, Y, Z*) can be projected into camera view *k* using the rotation matrix $R_{k}\in {\mathbb{R}}^{3\times 3}$, translation vector $t_{k}\in {\mathbb{R}}^{3}$, and intrinsic parameters $K_{k}\in {\mathbb{R}}^{3\times 3}$. The corresponding image coordinates (*u*_*k*_, *v*_*k*_) are obtained through the following equation:


(2) $$\begin{array}{l} \left(\begin{array}{c} u_{k}\;v_{k}\;1 \end{array}\right)={\text{K}}_{i}\times \left[{\text{R}}_{i}|{\text{t}}_{i}\right]\times \left(\begin{array}{c} X\;Y\;Z\;1 \end{array}\right) \end{array}$$

Next, we perform a min-pooling operation on the matrix of projected camera-view points *A* to generate a dense camera-view depth label *B*, with dimensions *m*×*n*, by downscaling by a factor of *k*:


(3) $$\begin{array}{r} B_{i,j}=\min\left\{A_{ki+p,kj+q}:p,q=0,1,\ldots ,k-1\right\}\\ \text{for }i=1,2,\ldots ,m\;\text{and }j=1,2,\ldots ,n \end{array}$$

We utilize focal loss as our depth loss function, which has the property of encouraging more evenly distributed softmax probabilities across multiple depth bins rather than concentrating on a single incorrect bin. This property is particularly useful when the model is uncertain about the depth, as distributing the context vector uniformly across depth bins helps mitigate the impact of incorrect predictions. Formally, we supervise the predicted depth distribution $\hat{\alpha }$ with the ground-truth depth distribution ***α***^***gt***^, where the depth loss is defined as:


(4) $$\begin{array}{c} L_{\text{Depth}}=-\sum {d=1}^{D}\alpha_{d}^{gt}\cdot {\left(1-{\hat{\alpha }}_{d}\right)}^{\gamma }\cdot \log\left({\hat{\alpha }}_{d}\right) \end{array}$$

In this formulation, $\alpha_{d}^{gt}$ is the one-hot encoding of the ground-truth depth class *d*, ${\hat{\alpha }}_{d}$ is the predicted probability for depth class *d*, γ is the focusing parameter, and *D* is the total number of discrete depth classes.

**Figure 2:**
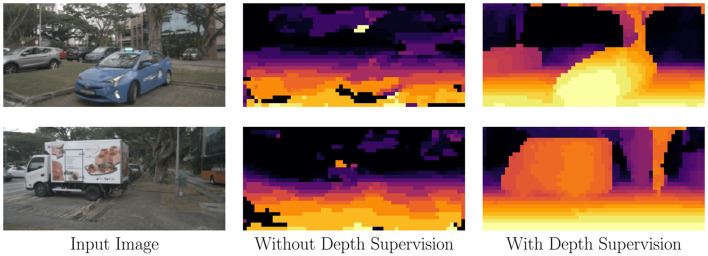
Qualitative results of the proposed camera-view supervisions. Supervising depth greatly improves the accuracy and meaningfulness of the predicted depth distribution.

As shown in Figure 2, the depth predictions from the “Without Depth Supervision” column are significantly inferior to those in the “With Depth Supervision” column. Furthermore, in Table 2, we observe that incorporating depth supervision results in a 30-fold reduction in depth prediction error. This substantial improvement demonstrates the necessity of depth supervision for accurate depth distribution predictions.

### 3.2 Segmentation supervision

Even with our proposed depth supervision, the depth prediction and by extension the mapping to BEV can still be noisy and incorrect. Semantic information is noisy and hard to decode correctly in BEV, leading to worse predictions. To address this, we propose supervising the extraction of camera-view features using ground-truth LiDAR points labeled with semantic segmentation classes. Specifically, we feed camera-view context features **c** into a segmentation head in order to predict a camera-view occupancy mask of relevant objects for each pixel. Although the output of the segmentation head is not directly used in the model, we show that supervising camera-view feature extraction teaches the model to extract more relevant features, which leads to better BEV performance. We can use the same process described in Section 3.1 to project a semantically labeled pointcloud into camera view. We adopt Binary Cross-Entropy loss to enforce relevant feature extraction in camera-view. Formally, we supervise camera-view context feature extraction by predicting binary camera-view occupancy mask **S**^*pred*^ with camera-view segmentation ground truth **S**^*gt*^. The loss that we use to supervise camera-view segmentation that we use can be formulated as


(5) $$\begin{array}{l} L_{\text{Seg}}=-\left({\text{S}}^{gt}\cdot \log\left({\text{S}}^{pred}\right)+\left(1-{\text{S}}^{gt}\right)\cdot \log\left(1-{\text{S}}^{pred}\right)\right) \end{array}$$

where **S**^*gt*^ is the ground truth camera-view segmentation and **S**^*pred*^ is the predicted probability. Camera-view segmentation supervision allows the model to more effectively learn relevant features in camera-view, which improves the feature quality in BEV frame.

### 3.3 Overall loss function

We use focal loss as our loss function in BEV. Our BEV loss can be defined as


(6) $$\begin{array}{l} L_{\text{BEV}}=-\left(y_{i}\cdot {\left(1-p_{i}\right)}^{\gamma }\cdot \log\left(p_{i}\right)+\left(1-y_{i}\right)\cdot p_{i}^{\gamma }\cdot \log\left(1-p_{i}\right)\right) \end{array}$$

where *y*_*i*_ is the true label of the BEV pixel *i*, *p*_*i*_ is the predicted probability of the BEV pixel *i*, and γ represents the focusing parameter of the loss. Our overall loss function involves three terms. The BEV, camera-view depth, and camera-view segmentation losses are summed together to create our final loss. We jointly optimize these terms during training. Our overall loss function is defined below.


(7) $$\begin{array}{l} L=L_{\text{BEV}}+\lambda_{\text{Depth}}L_{\text{Depth}}+\lambda_{\text{Seg}}L_{\text{Seg}} \end{array}$$

## 4 Implementation details

### 4.1 Architecture

For our camera-view backbone, we use a pre-trained EfficientNet-B4 (Tan and Le, 2019) and a camera input resolution of 224 × 480 for fair comparison to previous literature (Zhou and Krähenbühl, 2022; Hu et al., 2021; Chen et al., 2022). We downscale camera-view inputs by a factor of *k* = 8 from 224 × 480 to 28 × 60 to extract context features, following Hu et al. (2021). We discretize continuous camera-view depth into depth bins: $bin\left(d\right)=\left\lfloor \frac{d-2}{0.5}\right\rfloor +1,\;\text{for}2\leq d\leq 58$, which we find to be a good balance for the amount of depth bins. We implement the voxel-pooling method described in Liu et al. (2023) to perform our view transform from camera-view to BEV. We pool camera view features into a BEV grid of *C*×*H*× *W*, where *C* = 128, *H* = 200, *W* = 200, following previous literature (Hu et al., 2021). We utilize ResNet18 (He et al., 2016) as our BEV feature decoder to obtain our final prediction, following LSS and FIERY (Philion and Fidler, 2020; Hu et al., 2021). For our camera-view depth prediction module and camera-view segmentation prediction module, we use an architecture consisting of atrous spatial pyramid pooling and a deformable convolution layer to provide accurate depth estimation and occupancy masks.t This architecture allows us to handle geometric variations and multi-scale features (Chen et al., 2017). We use λ_Depth_ = 0.0025 and λ_Seg_ = 0.05, which we obtained through hyper-parameter tuning.

### 4.2 Training details

Following Zhou and Krähenbühl (2022), we train all models using focal loss (Lin et al., 2017) with γ = 2, following Zhou and Krähenbühl (2022). We use a batch size of 32 on 4 A6000 GPUs. We optimize using the Adam optimizer (Kingma and Ba, 2014) and the One-Cycle learning rate scheduler (Smith, 2017). We set the learning rate to 4 × 10^−3^ and the weight decay to 4 × 10^−7^, following Zhou and Krähenbühl (2022). We train for a total of 20 epochs, which finishes in approximately 6 h.

## 5 Experiments

Our experiments aim to show the effectiveness of our method and compare it to the state-of-the-art. We evaluate our method on vehicle and driveable region segmentation on a real-world commonly used driving dataset (Caesar et al., 2020).

### 5.1 Dataset

Following the evaluation settings of the previous baselines (Zhou and Krähenbühl, 2022; Philion and Fidler, 2020; Hu et al., 2021; Xu et al., 2022b; Chen et al., 2022), we evaluate our proposed method on the nuScenes dataset (Caesar et al., 2020). The nuScenes dataset comprises data from 1,000 real-world scenes, with each scene lasting 20 seconds and containing 40 frames. This results in a total of 40,000 samples. It offers a 360° view around the ego-vehicle through six camera perspectives, with each view providing both intrinsic and extrinsic details. We resize the camera images to 224 × 480 pixels, and generate Bird's-Eye-View (BEV) labels of 200 × 200 pixels for analysis. The dataset also contains semantically-labeled LiDAR points for each frame, which we use to generate camera-view depth and segmentation labels. Objects are annotated with 3D bounding boxes. Using the pose of the ego vehicle, we generate 200 × 200 BEV binary occupancy masks by orthographically projecting 3D bounding boxes to the BEV plane. We evaluate the quality of the bird's-eye-view segmentation in a 100*m*×100*m* region around the ego vehicle, and we sample the map at a 50 cm resolution. This setting was popularized by LSS (Philion and Fidler, 2020), and is used in most current literature (Zhou and Krähenbühl, 2022; Hu et al., 2021; Chen et al., 2022; Xu et al., 2022b).

### 5.2 Metrics

**Figure 3:**
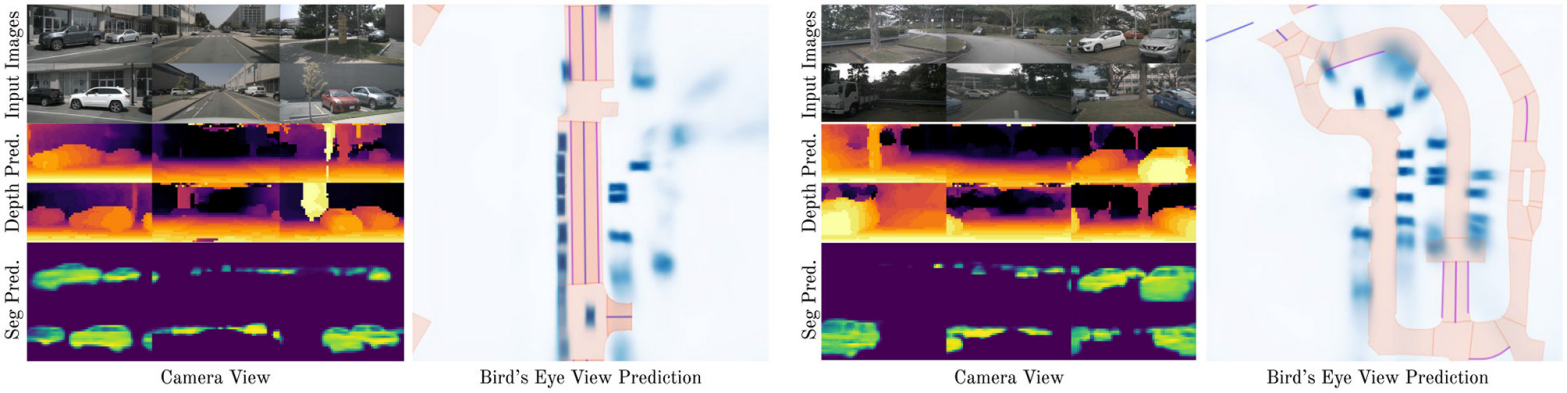
Qualitative results of our method. We show two examples, one on the left and one on the right. The leftmost column of each example shows camera-view images. The first row is the input image to the model, the second row is the camera-view depth prediction of our model, and the third row is the camera-view segmentation prediction of our model. The rightmost column of each example shows the segmentation prediction in bird's eye view. Our model meaningfully predicts camera-view depth and segmentation, and BEV segmentation.

We use Intersection-over-Union (IoU) to evaluate segmentation tasks, including bird's-eye-view and camera-view segmentation, following established literature (Philion and Fidler, 2020; Xu et al., 2022b; Hu et al., 2021; Zhou and Krähenbühl, 2022). IoU is a widely accepted metric that measures the overlap between the predicted and true regions. A higher scores indicates better performance. For camera-view depth prediction, we use Relative Square Error (RSE), as depth prediction involves predicting continuous values rather than binary labels (Xie et al., 2022; Eigen et al., 2014; Ranftl et al., 2021). A lower RSE score indicates better performance. We follow literature in using different metrics for segmentation (IoU) and depth prediction (RSE). Additionally, we report inference speeds on a single RTX 2080 Ti GPU and provide qualitative results in Figure 3. We report inference speeds with Frames Per Second (FPS), which is the amount of samples that our model can process per second.

### 5.3 Comparison to baselines

We compare our model to the most recent competitive benchmarks for real-time BEV semantic segmentation in Table 1. For a fair comparison we use models with a similar input resolution (in our case, 224 × 480), and only use camera information at test time. We also only use models that consider one single time-step prediction, since multi time-step prediction gives an advantage. We compare our method to VPN (Pan et al., 2020), OFT (Roddick et al., 2018), LSS (Philion and Fidler, 2020), CVT (Zhou and Krähenbühl, 2022), FIERY (Hu et al., 2021), CoBEVT (Xu et al., 2022b), and GKT (Chen et al., 2022). For vehicle segmentation, our method obtains a 3.8% higher IoU than the next most competitive model, GKT. For driveable region segmentation, our method obtains a 3.4% higher IoU than the next most competitive model that reports it. We also match previous baselines with a very competitive inference time of 32 FPS evaluated on a RTX 2080 Ti. As can be seen from Table 3, supervising camera-view depth greatly increases the accuracy of the depth projection, and since that depth projection is directly used in the camera-to-BEV projection, our method performs better. Table 3 also shows that our camera-view supervision also improves the feature extraction in camera-view. These factors contribute to the better performance of our method.

**Table 1:** Comparison to baselines on the nuScenes dataset.

**BEV model**	**Input resolution**	**Vehicle**	**Drivable area**	**Params (M)**	**FPS**
VPN	224 × 480	29.3	-	4	31
OFT	360 × 1, 080	30.1	71.7	-	-
LSS	128 × 352	32.1	72.9	14.3	25
FIERY	224 × 480	35.8	-	7.3	8
CVT	224 × 480	36.0	74.3	**1.1**	35
SinBEVT	512 × 512	37.1	-	1.6	35
GKT	224 × 480	38.0	-	1.2	**45**
**SUCAM (Ours)**	224 × 480	**41.8**	**78.1**	10.5	32

BEV Vehicle and Drivable Area evaluation is done with Intersection-over-Union (IoU). A score higher is better. Inference times are measured on an RTX 2080 Ti GPU. Our method performs at the state-of-the-art while maintaining competitive inference times. Best values are bolded.

### 5.4 Ablation study

In this section, we demonstrate the effectiveness of the components of our proposed method.

#### 5.4.1 Depth ablation study

Table 2 ablates different methods of obtaining the depth estimate used to project camera-view features into the BEV plane. The first row, Uniform Depth, is assigning a uniform depth distribution over all depths to each pixel. Note that there are no learnable parameters for this method. Soft Probabilities is what LSS (Philion and Fidler, 2020) and FIERY (Hu et al., 2021) use. It indirectly supervises the camera-view depth distribution of each pixel using the BEV segmentation loss. This method does involve learnable parameters, but there is no direct supervision. GT Depth directly uses ground-truth camera-view depth obtained from LiDAR point-clouds in order to project camera-view features to BEV. This method does not involve learnable parameters, because it directly uses ground-truth depth for the projection instead of predicting a distribution. Finally, $L_{\text{Depth}}$ directly supervises the depth distribution using ground-truth camera-view depth obtained from LiDAR point-clouds as the regression target.

**Table 2:** Ablation study of different depth projection methods.

**Depth type**	**BEV vehicle**	**Depth error**
Uniform depth	35.7	-
Soft probabilities	38.3	10.45
GT depth	**53.6**	-
$L_{\text{Depth}}$ (Ours)	40.9	**0.33**

BEV vehicle evaluation is done using Intersection-over-Union (IoU). Higher is better. Depth error is measured in Relative Squared Error (RSE), lower is better. We compare several representations of the camera-view depth distribution. Best values are bolded.

Based on Table 2, Uniform Depth performs the worst. We expect this because the model is unable to learn geometric relationships between objects in camera-view and BEV, and only is able to uniformly project features. Soft probabilities performs better because the model is able to use the final BEV segmentation loss to improve the depth distribution. As we expect, GT Depth performs the best by far, since it directly uses ground-truth depth to create an accurate projection of camera-view features to BEV. This provides motivation for us to supervise the depth distribution using the GT depth in $L_{\text{Depth}}$. We can see that by doing this, we can improve the IoU by 3% over the soft probabilities method. We can also see that this supervision causes a large reduction in the relative-squared-error (RSE) of the depth distribution by a factor of 30, proving that our depth supervision significantly improves the quality of camera-view depth estimation.

#### 5.4.2 Loss ablation study

We also ablate the effect of our method components in Table 3. We compare our base model, model with camera-view segmentation loss, model with camera-view depth loss, and model with both. For each method, we report the IoU of BEV segmentation, the predicted camera-view depth RSE, and the camera-view segmentation IoU (if applicable). Our base model achieves an IoU of 38.3%. Supervising camera-view segmentation improves this IoU by about 3% to 41.0% IoU, while achieving a camera-view IoU of 78.9%. Supervision of the depth distribution has a similar positive effect, improving IoU to 40.9%. Combining the two components improves IoU to 41.8%, an 5% improvement over the base model. We guess that the camera-view IoU decreases when adding depth loss because camera-view segmentation loss has a relatively small value when compared to the depth loss, and so outweighs it. This could be potentially solved with further hyper-parameter tuning. A future direction of work to improve the depth estimation even further would be to utilize multiple LiDAR sweeps from different time-steps to generate more dense labels.

**Table 3:** Ablation study of our proposed loss terms.

**Loss components**	**BEV vehicle**	**Depth error**	**Camera-view Seg**.
$L_{\text{BEV}}$	38.3	10.5	-
$L_{\text{BEV}}+L_{\text{Seg}}$	41.0	9.43	**78.9**
$L_{\text{BEV}}+L_{\text{Depth}}$	40.9	0.33	-
$L_{\text{BEV}}+L_{\text{Depth}}+L_{\text{Seg}}$	**41.8**	**0.27**	71.2

The first row is a model trained with only the base BEV loss. The second row is the BEV loss summed with the camera-view seg. loss. The third row is the BEV loss summed with the camera-view depth loss. The fourth row is our full model. Best values are bolded.

### 5.5 Model robustness

In this section, we evaluate the robustness of our proposed model in common real-world settings.

#### 5.5.1 Missing cameras

**Figure 4:**
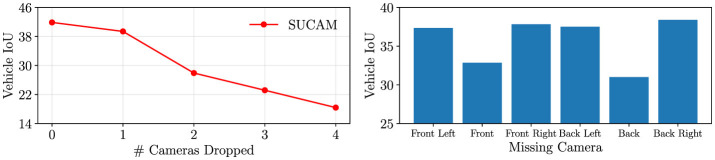
On the left, we measure model performance as we exclude *m* ∈ {0, 1, 2, 3, 4, 5} cameras from the input. On the right, we measure the performance of our model as we exclude specific cameras from the input.

A common real-world scenario in autonomous driving is dropped cameras: cameras that are disabled and go offline during operation. We evaluate the robustness of our model in this common situation. In Figure 4, we evaluate our model's performance as we exclude several cameras from our model input. The model's performance drops as the number of cameras decreases due to the decreased observed area. On the right, we measure the relative importance of each camera by testing model performance as we drop specific cameras from the input. The front and back cameras appear to be the most important, as the IoU decreases the most when these are dropped. Additionally, the back camera in nuScenes has the largest area covered out of all cameras, so as we expect, excluding this camera causes the largest IoU decrease. We note that in the most common case of one camera dropped, our model still maintains very respectable performance.

#### 5.5.2 Performance over distance

**Figure 5:**
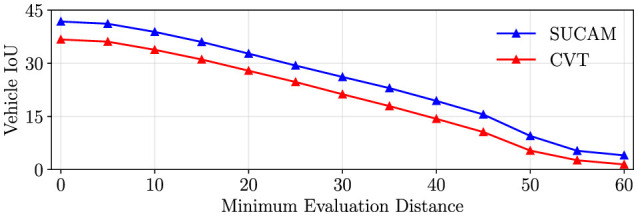
We measure the effect of distance on model performance. We measure the IoU as we increase the minimum distance from the vehicle for an object to be included in the IoU measurement. In other words, we exclude objects within the minimum distance from the ego vehicle from the IoU calculation. IoU drops approximately linearly with the minimum distance of evaluation. This may be because objects that are farther away are more prone to incorrect camera to BEV projections.

Figure 5 illustrates the robustness of our method as the distance of perceived objects from the ego vehicle increases. We measure IoU while varying the minimum distance threshold for objects to be included in the IoU computation. Specifically, for each minimum distance value, we exclude any object closer than the specified threshold to the ego vehicle during the IoU calculation. This analysis allows us to assess how well our method performs in segmenting objects at different distances from the ego vehicle. We observe the impact of object distance on the segmentation accuracy of our method (depth-based projection) and CVT (transformer based projection). IoU drops approximately linearly with the minimum distance of evaluation for both methods. This may be because objects that are farther away are more prone to incorrect camera to BEV projections, regardless of the projection method. Performance is expected to decrease over distance for a perception model. In real-world scenarios, predictions that are a large distance away from the sensors should be trusted less. A future direction of work may be to quantify this predictive uncertainty.

## 6 Conclusion

Bird's eye view (BEV) semantic segmentation is crucial for autonomous vehicles to accurately perceive and understand their surroundings in real-world driving scenarios. In this work, we introduce two novel supervision processes that significantly enhance the performance of real-time BEV semantic segmentation: camera-view depth supervision and camera-view segmentation supervision. Camera-view depth supervision helps our approach better capture spatial relationships and depth information, leading to more accurate camera-to-BEV mappings. Camera-view segmentation supervision allows the model to more effectively learn the semantic correlations between camera-view and the BEV frame. Some limitations of our work are the fact that our training scheme requires LiDAR data and camera-level labels during testing time. Our camera-view depth and segmentation supervision also adds extra computational cost to the overall model. Note that our model still only needs RGB camera inputs during inference. Through extensive qualitative and quantitative evaluations, we demonstrate that our proposed method shows significant improvement compared to previous methods. We also run experiments to show the robustness of our methodology to common real world scenarios such as dropped cameras and distant objects.

## Data Availability

The original contributions presented in the study are included in the article/supplementary material, further inquiries can be directed to the corresponding author.
